# Supplementary material for: Identification of photoperiod-induced specific miRNAs in the adrenal glands of Sunite sheep (Ovis aries)
Source: Front Vet Sci. 2022 Jul 22;9:888207. doi: 10.3389/fvets.2022.888207 (PMC9354845; doi:10.3389/fvets.2022.888207)
Supplement: Supplementary file 1 [file Data_Sheet_1.ZIP › Supplementary file/miRDeep2 file.zip!/miRDeep2 file/Know_Structure/SPLP42A3/oar-mir-133.pdf]

miRBase precursor : oar-mir-133
Total read count : 151
oar-miR-133 read count : 144
remaining reads : 7

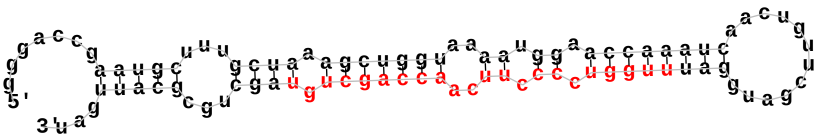

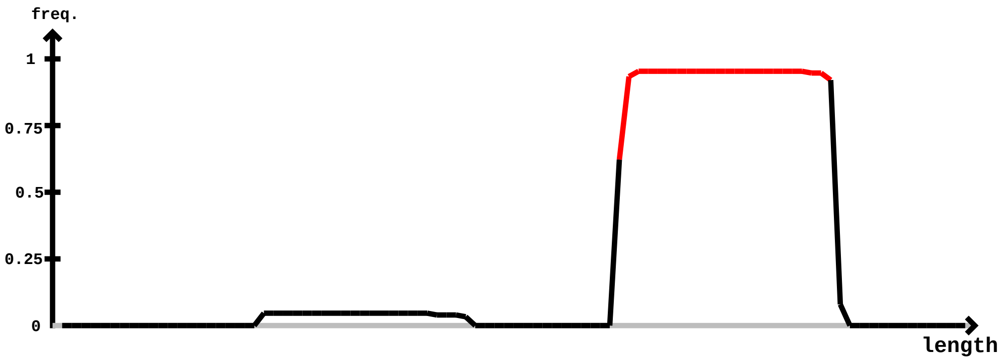

oar-miR-133

```
5'- gggaccgaaugcuuugcuaaagcugguaaaauggaaccaaaucaacuguucgauggauuugguccccuucaaccagcuguagcugcgcauugau   -3'    exp
    .......(((((...((((.(((((((..((.((.((((((((............)))))))).)).))..))))))).))))...)))))...    reads    mm    sample
    ....................agcugguaaaauggaacc........................................................    1        0     seq
    ....................agcugguaaaauggaaccaaa.....................................................    1        0     seq
    ....................agcugguaaaauggaaccaaau....................................................    5        0     seq
    .........................................................uuugguccccuucaaccagcug...............    4        0     seq
    .........................................................uuugguccccuucaaccagcugu..............    84       0     seq
    .........................................................uuugguccccuucaaccagcugua.............    6        0     seq
    ..........................................................uugguccccuucaaccagc.................    1        0     seq
    ..........................................................uugguccccuucaaccagcugu..............    40       0     seq
    ..........................................................uugguccccuucaaccagcugua.............    6        0     seq
    ...........................................................ugguccccuucaaccagcugu..............    3        0     seq
```
